# Oxygen-Cluster-Modified Anatase with Graphene Leads to Efficient and Recyclable Photo-Catalytic Conversion of CO_2_ to CH_4_ Supported by the Positron Annihilation Study

**DOI:** 10.1038/s41598-019-49694-w

**Published:** 2019-09-11

**Authors:** Gulzar Ahmed, Fazal Raziq, Muddasir Hanif, Javid Khan, Khurram Shahzad Munawar, Mingmei Wu, Xingzhong Cao, Zhongwu Liu

**Affiliations:** 1 School of Materials Science and Engineering, South China University of Technology, Guangzhou, 510640 P.R. China; 2 School of Physics, University of Electronic Science and Technology of China, Chengdu, 610054 P.R. China; 3 State Key Laboratory of Luminescent Materials and Devices, South China University of Technology, Guangzhou, 510640 P.R. China; 4 MOE Key Laboratory of Bioinorganic and Synthetic Chemistry, School of Chemistry and Chemical Engineering, Sun Yat-Sen University, Guangzhou, 510275 P.R. China; 5 University of Sargodha Sub Campus Mianwali, 42200 Punjab, Pakistan; 6 Institute of High Energy Physics, Chinese Academy of Sciences, Beijing, 100049 P.R. China

**Keywords:** Carbon capture and storage, Pollution remediation

## Abstract

Anatase TiO_2_ hollow nanoboxes were synthesized and combined with the graphene oxide to get nanocomposite of TiO_2_/rGO (TG). Graphene oxide was used to modify the Oxygen-Clusters and bulk to surface defects. Anatase and TG composite were characterized with the positron annihilation, XPS, EPR, EIS and photocurrent response analysis. The relative affects of defects on the photocatalytic reduction (CO_2_ to CH_4_) were studied. The TG composites showed highest photo-catalytic activity after GO coupling (49 µmol g^−1^ h^−1^), 28.6 times higher photocurrent yields much higher quantum efficiency (3.17%@400 nm) when compared to the TiO_2_ nanoboxes. The mechanism of enhanced photo-catalytic CO_2_ conversion to CH_4_ elucidated through electrochemical and photo-catalytic experiments with traceable isotope containing carbon dioxide (^13^CO_2_). For the first time we discovered that diminishing the comparative concentration ratio of anatase from the bulk to surface defects could significantly increase the conversion of CO_2_ to CH_4_.

## Introduction

The carbon dioxide (CO_2_) emission is one of the serious environmental problems, therefore its reduction and sustainable conversion to fuels by using solar energy is highly desirable^1^. Titania (TiO_2_) is a chemically stable and biologically benign photo-catalyst, capable to convert harmful pollutants, particularly CO_2_ reduction to fuel CH_4_^2^. Beside the well-known bulk defects, the surface defects are more sensitive to the photocatalytic activities. The surface defects in a pure crystal can act as e-h trap-sites along with the adsorption sites on the other hand the bulk defects act only as charge carrier traps where e-h are more likely to recombine. Therefore, there is great scientific importance to understand both the bulk and surface defect states. This study highlights the defect density effects on the photocatalytic activities, which may lead to the innovative design of photocatalysts. The defects in TiO_2_ have been experimentally studied by the scanning tunneling microscopy (STM)^3^, electron paramagnetic resonance (EPR)^4^, time-resolved photoluminescence spectroscopy (PL)^5^ etc. These techniques allow understanding the effects of surface or subsurface defects, thereby helped to correlate the adsorption and surface reactivity.

The surface of graphene oxide consists of oxygen and hydroxyl functional groups capable to form new bonds with the TiO_2_. When combined with graphene, the composite provides an effective approach to improve the photo-catalytic efficiency and light response. This occurs because graphene layers with 2D sp^2^-hybridized carbon atoms (pi-bond conjugated structure) have superior electronic properties and large specific surface area that significantly enhances the photo-catalytic properties^6,7^. Compared with the other defect-probes techniques, positron annihilation lifetime spectroscopy (PALS) is a non-destructive technique with many advantages. For example, PALS is capable to probe not only surface but also bulk defects, sensitive to all kinds of defects particularly defects with complex structures such as open volume porousity, aggregates, voids, disclocations and grain boundaries. Therefore, PALS is a good addition to the XPS, EPR and PL techniques.

Herein, TiO_2_ nano-boxes with bulk/surface defects were synthesized and characterized with the positron annihilation. We found that lowering the relative bulk to surface defects concentration ratio improved the photocatalysis efficiency. We report exceptionally enhanced photocatalytic CO_2_ reduction capability of titania through defects and structural engineering, as well as magnetic titania hybrid for efficient recyclability. Simple routes were developed to synthesize the nano-crystalline anatase TiO_2_ with hollow structure of nano-boxes. For the defect engineering and photocatalytic properties, nanocomposites of TiO_2_/rGO (TG) and TiO_2_-(Fe_3_O_4_@SiO_2_)/rGO (TFSG) hybrids were synthesized through controlled microwave processing strategy. To improve the recyclability, TFSG samples with Fe_3_O_4_ magnetic nano-core and SiO_2_ protecting shell were synthesized for the recovery of catalyst. Herein we discuss our findings in detail.

## Results and Discussion

### Structural characterization and surface composition

The XRD spectra of TiO_2_, and TG composite (Supplementary Fig. 1a(i)) indicates that the samples have crystalline nature. The diffraction peaks indexed as (101), (004), (200), (105), (211), (204), (116), (220), (215) and (224) for the anatase phase matched well with the reference (JCPDS: 01-071-1166), indicating a single phase. The obtained TG sample shows TiO_2_ peaks because rGO is not sensitive to the XRD (Supplementary Fig. 1a(ii))^8^. The Raman spectra of composites (Supplementary Fig. 1b) exhibit typical features of rGO with D-band at 1354 cm^−1^ and G-band at 1611 cm^−1^. The remaining four peaks from 100–700 cm^−1^ belong to the TiO_2_. When the geometric size of TiO_2_ crystal approaches to nanometer range, the *E*_g_ Raman active mode blue-shifts to 150 cm^−1^ (instead of 143 cm^−1^) for the bulk phase^9^. The other specific vibration modes at 400 cm^−1^ (B_1g_), 523 cm^−1^ (B_1g + _A_1g_) and 643 cm^−1^ (*E*_g_) are characteristic peaks of the anatase phase.

**Figure 1:**
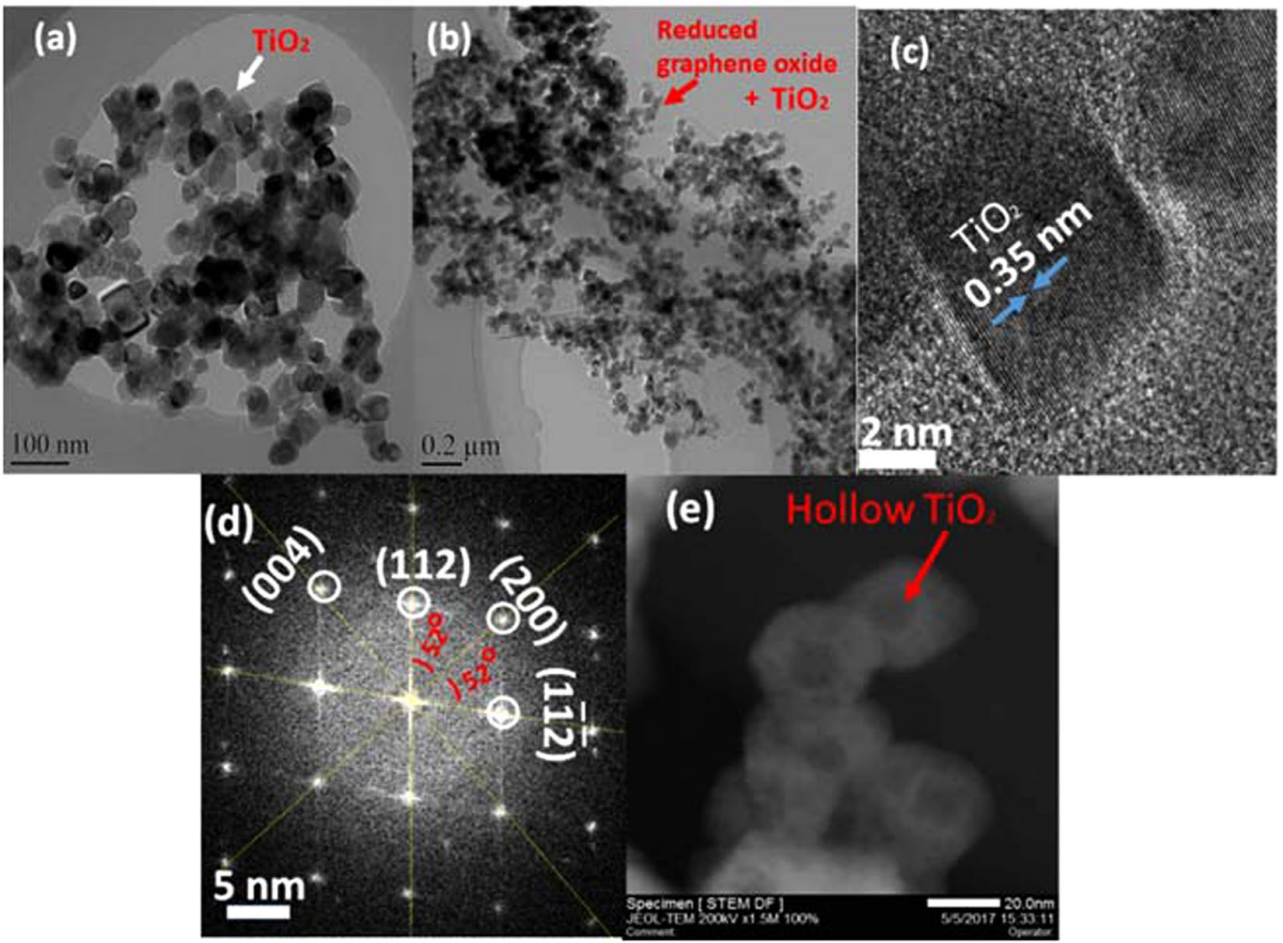
Characterization of TiO_2_ hollow nanoboxes and TG composite. (**a**) Bright-field TEM images of the hollow TiO_2_ nanoboxes. (**b**) TEM of TiO_2_@rGO (TG composite). (**c**) The HRTEM shows the lattice spcing 0.35 nm (101). (**d**) corresponding SAED pattern. (**e**) STEM of hollow TiO_2_.

The C1s XPS spectra indicated the existence of chemical binding between TiO_2_ and rGO (details in Supplementary information D1). Compared with the blank GO, the new peak at 285.5 eV is ascribed to the Ti-O-C bonding. Therefore there is covalent bonding between the TiO_2_ and rGO. This is further confirmed by the significnt decrease in the FTIR peks at 454, 668 and 803 cm^−1^. The bright-field TEM (Fig. 1a) of TiO_2_ indicated hollow box like structures with narrow size distribution and good morphological uniformity. The TiO_2_ nanoboxes on graphene sheets (Fig. 1b) showed well dispersed NPs on both the graphene sides. The reduced graphene oxide is transparent with wrinkles and hollow TiO_2_ structures (HRTEM, lattice spacing of 0.35 nm, Fig. 1c), confirming the (101) planes of the anatase TiO_2_. The SAED pattern (Fig. 1d) revealed a [010]-axis orientation, and the lateral plane of the of anatase corresponds to {010} facet that is vertical to the [010] orientation. The Fig. 1e STEM image shows hollow TiO_2_ nanoboxes.

### Defect characterization by PALS, XPS and EPR

**Figure 2:**
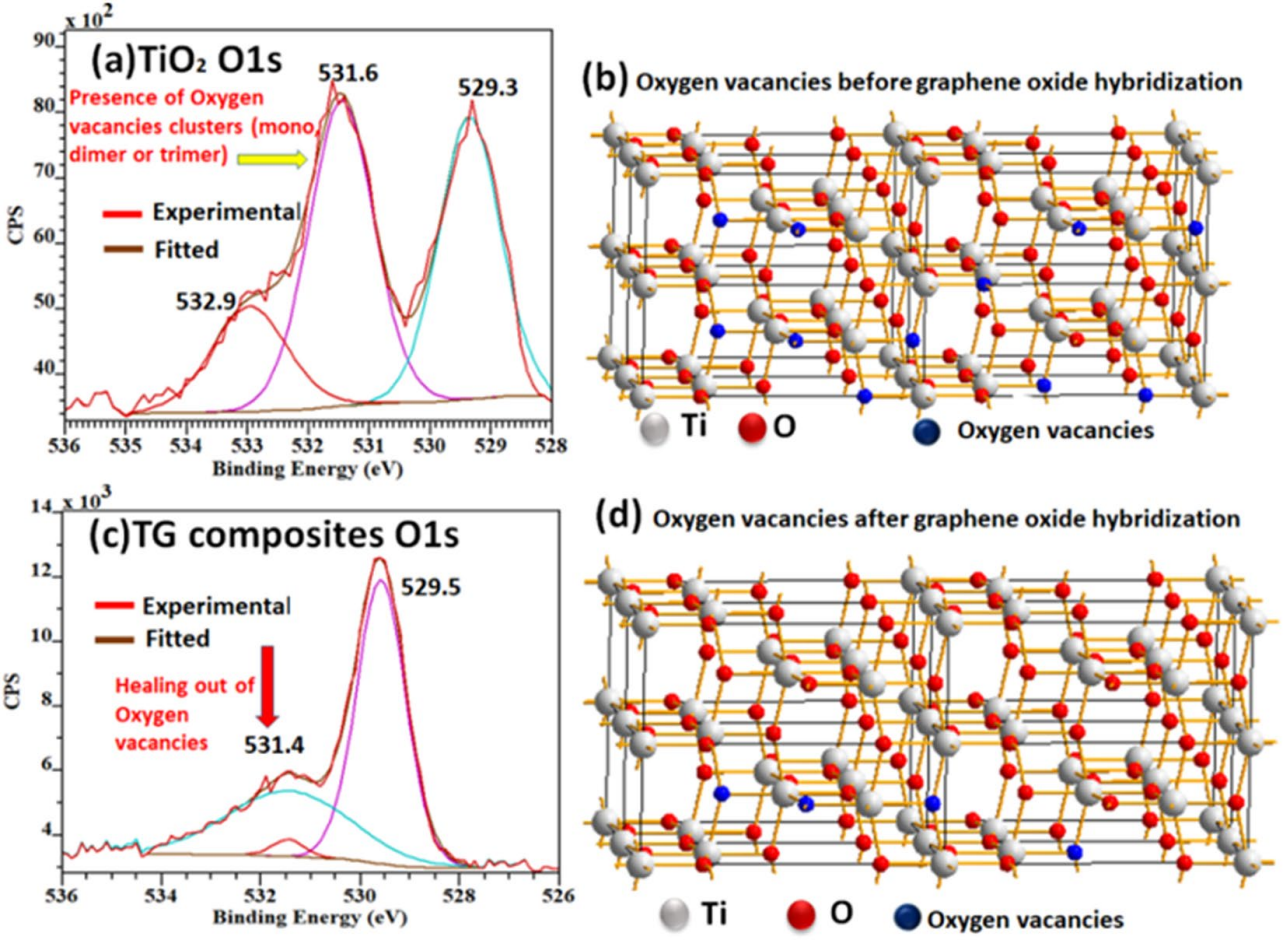
(**a**) XPS spectrum (TiO_2_) of measured O1s spectrum, (**b**) simulated oxygen vacancies in 2*2-anatase crystal of the TiO_2_ nanoboxes. (**c**) Measured XPS O1s spectrum of the TG composites. (**d**) Healing of the oxygen vacancies in the 2*2-anatase crystal.

Previous reports showed oxygen defects on the surface of TiO_2_ raise the local-states under the edge of the conduction band. Oxygen defects densities with one or two electrons are generated due to the missing oxygen atom in the bulk or on the surface, while the Carbon doped TiO_2_ forms deep impurity states in the band gap^1–3,10,11^. We observed (UV-vis spectrum, Supplementry Fig. 2) that bandgap of TiO_2_ (3.2 eV) significantly decreased (2.6 eV) with increased absorption after hybridization with GO.

In this section, we used PALS (positron annihilation lifetime spectroscopy) to quantify the defect concentration obtained by the comparison of TiO_2_ and TG composites. The Table 1 shows three positron lifetime components, τ_1_, τ_2_, and τ_3_, with relative intensities I_1_, I_2_, and I_3_ for the TiO_2_ nanoboxes and TG composites. The shortest positron lifetime τ_1_ (0.1985 ns) can be assigned to the bulk defects with free annihilation of positrons inside defect-free crystal^12^. Where as the longer lived positrons τ_2_ (0.3937 ns) can be attributed to the surface defects originating from the positrons trapped by the larger defects such as oxygen vacancies or clusters, homodimers, trimers or larger^10^. The second positron lifetime components (τ_2_) for the TiO_2_ nanoboxes and TG composites are much higher than their respective first positron lifetime components (τ_1_). The variation of τ_2_ value from 0.3937 ns to 0.3864 ns was evident that the TiO_2_ nanoboxes contain an abundance of surface oxygen defects and compressive bonding but reduced to atomic dimensions in the TG composites. The largest component (τ_3_) is assigned to the micropores and to the annihilation of orthopositronium atoms^11^. Besides the lifetime factor of the positron, its relative intensity (I) gives information regarding the relative concentration of the defects^13^. The coupling of graphene oxide with TiO_2_ leads to the decreased I_1_/I_2_ (1.23 to 1.03), thereby clearly indicates the relative bulk to surface defects concentration decrease.

**Table 1:** Positron life time and relative intensities of the TiO_2_ and TG sample

Sample	τ_1_ (ns)	τ_2_ (ns)	τ_3_ (ns)	I_1_ (%)	I_2_ (%)	I_3_ (%)	I_1_/I_2_
TiO_2_	0.1985	0.3937	2.29	54.38	43.86	1.763	1.23
TG	0.2069	0.3864	2.313	50.1	48.3	0.669	1.03

The samples were further investigated by the XPS to get information about the chemical composition, oxygen vacancies and Ti oxidation-state inside TiO_2_ nano-boxes after microwave treatment with the rGO. The O1s spectrum of TiO_2_ nanobox (Fig. 2a) shows a peak at the binding energy of 529.3 eV, corresponding to the Ti(IV) bound oxygen (O^2−^). The peak at 531.6 eV can be attributed to the high binding energy component originated from the loss of oxygen (oxygen vacancies)^14^. The peak at 532.9 eV (low binding energy component), is due to the adsorption of HO^−^ on the surface. The O1s spectrum of TG composite (Fig. 2c) showed a strong O^2−^ peak at 529.5 eV. The second peak related to the oxygen vacancies O* (531.6 eV), showed significant suppression after rGO hybridization (microwave treatment). A slight shift (0.2 eV) in the peak position (529.5 eV) for the oxygen anions (Ti–O–Ti) is consistent with the Ti 2p core-level due to hybridization with graphene. Furthermore, inside TG sample the third low energy peak (near 532.9 eV) related to the surface hydroxyl group almost vanished. These observations confirmed that the concentration of surface defects is related with both surface hydroxyl groups and oxygen vacancies were significantly lowered in the hybrid sample^15^. The Fig. 2b,d schematically illustrates oxygen vacancies before and after GO hybridization.

**Figure 3:**
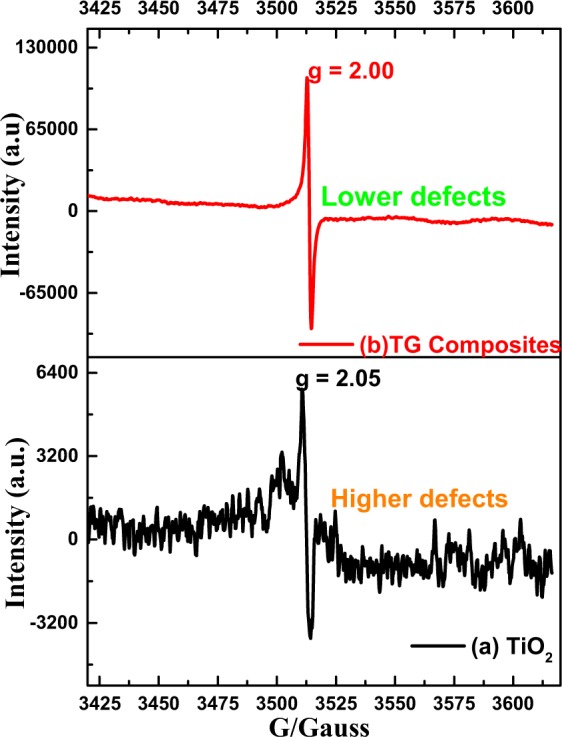
(**a**) EPR spectra of the TiO_2_ and (**b**) TG composite at room temperature.

The defects were further analyzed by the EPR (Fig. 3) because of the sensitivity of EPR towards the different defects, caused by the lower or extra electrons present on different atoms (O^−^, O^2−^, Ti^3+^, Ti^4+^) under different surrounding atoms (spin Hamiltonian, perturbation theory). The EPR spectra for the TiO_2_ shows several peaks beside the main peak (g = 2.05). This observation clearly indicates presence of several types of defects. The previously reported EPR spectra indicated that the initial hole-trapper at the lattice of oxide ions at hydrated TiO_2_ surfaces are the hydroxyl radicals. These trapped holes are the defect centers termed as bridging O^−^ species^16^. The presence of signal at g = 2.05 with broad underline peak in TiO_2_ nanobox (Fig. 3a) represents the existence of significant oxygen vacancies^17^. This occurs because electrons trapped at Ti^4+^ sites can form Ti^3+^ (XPS, Fig. 2) and holes at subsurface oxide ions can form O^**−**^^18^. The obtained EPR spectrum for the TG composites (Fig. 3b) shows a strong paramagnetic signal at g = 2.00 with loss of extra sharp-peaks. This represents the healing of oxygen vacancies (smoother spectrum) and other defects. Therefore we concluded that rGO hybridization has remarkably lowered the oxygen vacancies and other defects. These observations were further tested by the hydrofluoric acid (HF) addition experiments. Defects were introduced into TiO_2_ by changing the amount of HF (details in Supplementary Information D2). The comparison showed defect introduction by the HF (0, 50 µL) changed the shape of EPR, introduced extra peaks (less smooth EPR) and modified the g value of TiO_2_.

### Absorption

The UV-Vis spectra of TiO_2_ nano-boxes and composites (Supplementary Fig. 2) exhibit extended absorption therefore indicates lowered band gap of composites when compared with the standard TiO_2_. The decrease in band gap has been attributed to the formation of C-Ti bond^19^. It is well-known that the extended absorption of light for the TG composite opens the possibility of higher photo-catalytic activity of a given photo-chemical reaction. Furthermore, considering the practical applications, the recyclability of photo-catalyst is another challenge to avoid contamination and higher economic efficiency. The photo-catalyst composites containing magnetite can easily separate from the solution by an external magnet. Therefore, the designing of efficient, magnetic photo-catalytic composite with reliable and stable structure has high importance. The non-toxic SiO_2_ coating layer, conjugates well with functional groups, and known to protect Fe_3_O_4_ from oxidation thereby increases stability of NP-decorated photo-catalyst. Therefore, we have incorporated these materials to construct our photo-catalysts (TiO_2_-Fe_3_O_4_@SiO_2_)@graphene TFSG composite. The percentage of nanocomposites was TiO_2_ (85%), Fe_3_O_4_@SiO_2_(5%) and graphene (10%). The XRD spectra of TiO_2_, Fe_3_O_4_@SiO_2_ and (TiO_2_-Fe_3_O_4_@SiO_2_)@graphene TFSG composite (Supplementary Fig. 3) indicates that all the samples are crystalline in nature. The obtained TFSG sample showed both peaks of TiO_2_ and Fe_3_O_4_@SiO_2_ nanoparticles (Supplementary Fig. 3(iii)), thus confirming the composite nature. The HRTEM of composites showed uniformely coated Fe_3_O_4_ with SiO_2_ and and TiO_2_ (Supplementary Fig. 4a,b).

The comparison of magnetic hysteresis loops related to the Fe_3_O_4_, Fe_3_O_4_@SiO_2_ and TFSG composites (Supplementary Fig. 5) showed room-temperature ferromagnetism for the Fe_3_O_4_ and Fe_3_O_4_@SiO_2_ and TFSG composites. The results show that there is a decrease in the magnetic saturation of TFSG composite due to the non-magnetic TiO_2_. The saturation magnetization value (M_s_) of the TFSG composites is 3.18 emu g^−1^, which is lower than those of the Fe_3_O_4_@SiO_2_ (57.48 emu. g^−1^) and Fe_3_O_4_ (64.03 emu. g^−1^). This value of magnetization is good for the efficient recovery of photo-catalyst.

### Photocurrent and charge separation

**Figure 4:**
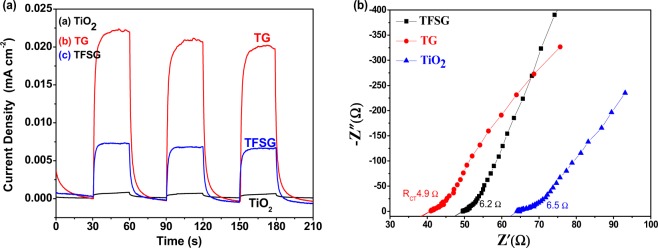
(**a**) I–t curves for the photocurrent responses of the TiO_2_ nanoboxes (a), TG (b) and TFSG composites (c). (**b)** The Nyquist plot for the TiO_2_ nanoboxes (a), TG (b) and TFSG composites (c).

The transient photocurrent response for the TiO_2_, TG and TFSG composites were compared (Fig. 4a). The photocurrent (photo-electrochemical) response of the TiO_2_ nanobox was weak (8.2 × 10^−5^ mA.cm^−2^). While the TG composite showed significantly increased photocurrent response 2.344 × 10^−2^ mA.cm^−2^ (28.6 times higher) and 9.786 × 10^−4^ mA.cm^−2^ for the TFSG composites (11.96 times higher). This significant photo-current enhancement attributed to the lower recombination rate of electron–hole pairs and longer lifetime due to the presence of rGO sheets (2D 𝜋-𝜋 conjugation structure) as compared to the TiO_2_. The comparison of EIS curves (Fig. 4b) showed the single-semicircles (Nyquist plots) in the high frequency range and a linear behavior in the low frequency region. The lowest charge transfer resistance (*R*_ct_) was observed (4.9 Ω) for the TG composite when compared to the TFSG (6.2 Ω), and TiO_2_ (6.5 Ω). Therefore, the TG composite exhibits the lowest *R*_ct_, indicating lower charge-transfer resistance resulting in much faster separation of photo-generated species^20,21^.

To verify the carbon detected in CO_2_ conversion originates from the decomposition of photo-catalyst or from the added CO_2_ gas, we carried out isotopic experiments by using traceable ^13^CO_2_ under identical photo-catalytic reaction conditions. The products of photo-catalytic reaction were identified and quantified by the GC-MS (Supplementary Fig. 7). The ^13^C, ^13^CH, ^13^CH_2_, ^13^CH_3_ and ^13^CH_4_ molecular fragments were detected. These observations from MS spectrum confirmed that the produced CH_4_ indeed originated-from the reduction of CO_2_ gas and not from the photocatalyst decomposition.

### Photo-catalytic activities

**Figure 5:**
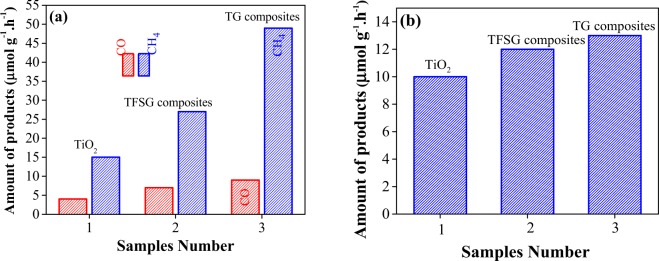
(**a**) Photocatalytic conversion of CO_2_ to CH_4_ after 1 h, (**b**) H_2_ production by the photocatalysis (UV-Vis irradiation) of TiO_2_ nanoboxes, TG composites and TFSG composites.

The photo-catalytic activities of TiO_2_ nanoboxes, TG composites and TFSG composites samples were evaluated for CO_2_ conversion with triethanol amine as the electrons donor (Fig. 5a). The TiO_2_ nanoboxes showed weak response for the photo-catalytic CO_2_ conversion to CH_4_ (~15.0 µmol g^−1^ h^−1^) after 1 h irradiation. The TG composites demonstrate the highest (~49.0 µmol g^−1^ h^−1^) activity for CO_2_ conversion. Since, the TFSG composites were hybridized with magnetic nanoparticles (Fe_3_O_4_@SiO_2_) for easier recovery thereby it showed lower CO_2_ conversion to CH_4_ activity (27.0 µmol g^−1^ h^−1^) than TG, but still 3.2 times higher than that of TiO_2_ nanoboxes (15.0 µmol g^−1^ h^−1^). The main detected gas products were CH_4_, H_2_ and CO (GC analysis). The Fig. 5b shows the quantitative comparison of produced H_2_ during this experiment. We further performed blank tests to confirm that CH_4_ comes from the reduction of CO_2_. After blank photo-catalytic experiment without CO_2_ under the same conditions, we have not detected any CH_4_.

**Figure 6:**
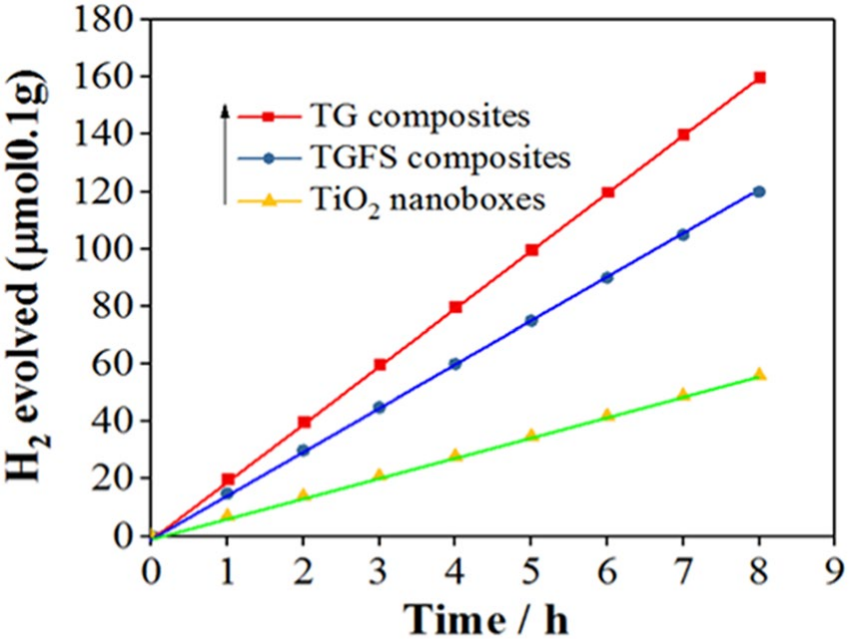
Time courses of H_2_ evolution resulting from the reaction of water by TiO_2_ nanoboxes, TG composites and TFSG composites irradiated with UV-visible light.

The Fig. 6 shows comparison of time dependent H_2_ evolution for the TiO_2_, TG and TFSG composites. This water splitting reaction showed a sustained H_2_ release rate of ~18.46 µmol/h for the TG composites. The standard solar light illumination for 8 hours, the hydrogen evolution kept increasing, demonstrating the robust photo-catalytic performance. This observation indicates stable and active photo-catalyst for long term water splitting. After conversion of CO_2_ to CH_4_ (5 h), samples were collected, washed several times and dried. The TEM results (Details in Supplementary information D3) showed stable composite after 5 h light irradiation.

The rate of H_2_ evolution followed the following order: TG composites > TFSG composites > TiO_2_ nano-boxes respectively. Quantum efficacies of these samples were calculated (Supplementary information 8) as 3.02%, 2.01%, and 0.3%, respectively for the TG, TFSG, and TiO_2_ nanobox. In the CO_2_ reduction, the photo-generated electrons-holes would migrate to the rGO surface. The conduction band of TiO_2_ nano-boxes and the work function of rGO are reported as −4.2 eV and −4.42 eV, respectively^22,23^. The proximity of energy levels is helpful for the photo-generated species transfer from the TiO_2_ nano-boxes conduction band to the rGO thereby produces radicals. The reason behind is the adsorption kinetics of the CO_2_ (10^−8^ to 10^−3^ s) on TiO_2_ nanoboxes is slower than the e-h recombination time (10^−9^ s)^24,25^. The composites increased the lifetime of the charged species that further improved the selective formation of CH_4_ gas along with other minor product gases. The photo-catalytic performance supports our proposed interpretations of the CO_2_ conversion to CH_4_ through reduction mechanism.

**Figure 7:**
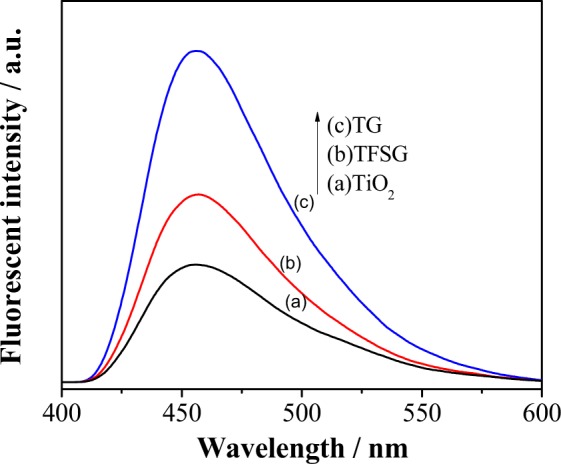
The ^•^OH radical amount-related fluorescence spectra of (**a**) TiO_2_ nanoboxes, (**b**) TFSG and (c). TG composites.

As reported the hydroxyl radicals (·OH) are recognized to be important intermediates for CH_4_ conversion. The mechanism of charge separation, coumarin fluorescence method (coumarin + ^•^OH → fluorescent 7-hydroxycoumarin) is used to detect the amount of hydroxyl radicals (^•^OH) produced via photochemistry. The procedure relies on the basic principle that: the stronger the fluorescence signal being observed, the larger the ^•^OH produced. We further compared the produced ^•^OH for TiO_2_ nanoboxes, TFSG and TG composites. The amount of produced ^•^OH in TG and TFSG is larger than the TiO_2_ nanoboxes (Fig. 7), with TG composites showing the highest fluorescence intensity. Hence, the TG composites produced highest amount of ^•^OH thereby confirming the excellent photo-electrochemical and photo-catalytic properties.

In summary, we have explored the photo-catalytic reduction of CO_2_ on TiO_2_ nano-boxes, TiO_2_ hybridzed with graphene (TG) and TiO_2_-(Fe_3_O_4_@SiO_2_)-rGO nanocomposite (TFSG). Oxygen clusters along with surface/bulk defects in TiO_2_ nanoboxes play vital role in efficient photocatalysis. Positron annihilation results revealed decreased Oxygen clusters (mono, dimers, trimers) and the relative concentration ratio of bulk/surface defects in TiO_2_ nanoboxes efficiently enhanced the photocatalytic mechanism. The photo-catalytic experiments and traceable isotopes containing carbon dioxide (^13^CO_2_) analyzed through mass spectroscopy confirmed the source of CH_4_ production. The TG composite exhibits 3.2 times (~49.0 µmol g^−1^ h^−1^) higher photo-catalytic reduction of CO_2_ to CH_4_, when compared to the TiO_2_ nanoboxes. The TFSG composites retained magnetization, therby provides an easy way for the catalyst recyclability. The XPS study revealed that number of oxygen vacancies significantly decreased after GO hybridization, further confirmed by the EPR result. The excellent electrochemical and photo-catalytic properties were assigned to the lowered defects, proximity of energy levels, much higher photo-current and higher quantum efficiency (3.17% @400 nm). We have presented 4 important evidences (positron annihilation (I_1_/I_2_ lowered), XPS (peaks 532, 531 eV decreased), EPR (smoothed, enhanced-signals), EIS (lowered charge-transfer resistance *R*_CT_) confirming that the GO modified the TiO_2_ to get appropriate photo-catalytic properties. Further by comparison and appreciation with the recent research^26–31^ we believe, this research highlights the importance of meticulous design of the photo-catalysts required to improve the selective conversion of CO_2_ towards valuable fuels^32^.

## Supplementary information

Supplementary Info

## Data Availability

The data sets generated during and/or analysed during the current study are available from the corresponding authors on reasonable request.
