# Supplementary material for: Oxygen-Cluster-Modified Anatase with Graphene Leads to Efficient and Recyclable Photo-Catalytic Conversion of CO2 to CH4 Supported by the Positron Annihilation Study
Source: Sci Rep. 2019 Sep 11;9:13103. doi: 10.1038/s41598-019-49694-w (PMC6739302; doi:10.1038/s41598-019-49694-w)
Supplement: Supplementary file 1 — Supplementary Info [file 41598_2019_49694_MOESM1_ESM.docx]

Oxygen-Cluster-Modified Anatase with Graphene Leads to Efficient and Recyclable Photo-Catalytic Conversion of CO_2_ to CH_4_ Supported by the Positron Annihilation Study

Gulzar Ahmed^a,e^*, Fazal Raziq^b^, Muddasir Hanif^a,c^*, Javid Khan^d^, Khurram Shahzad Munawar^e^,

Mingmei Wu^d^, Xingzhong Cao^f^, Zhongwu Liu^a^*

^a^School of Materials Science and Engineering, South China University of Technology, Guangzhou 510640, P. R. China

^b^School of Physics, University of Electronic Science and Technology of China, Chengdu 610054, P. R. China

^c^State Key Laboratory of Luminescent Materials and Devices, South China University of Technology, Guangzhou 510640, P. R. China

^d^MOE Key Laboratory of Bioinorganic and Synthetic Chemistry, School of Chemistry and Chemical Engineering, Sun Yat-Sen University, Guangzhou 510275, P. R. China

^e^University of Sargodha Sub Campus Mianwali 42200, Punjab, Pakistan

^f^Institute of High Energy Physics, Chinese Academy of Sciences, Beijing 100049, P. R. China

*E-mail: [gulzar.ahmed@uos.edu.pk](mailto:gulzar.ahmed@uos.edu.pk) (G. Ahmed), muddasirhanif@yahoo.com (M. Hanif), [zwliu@scut.edu.cn (Z.W](mailto:zwliu@scut.edu.cn(Z.W). Liu)

**Supplementary Fig .1a and 1b**

**Supplementary Fig. 1 a and b| a (i, ii)**, XRD pattern of TiO_2_ and TG composites, which preferentially shows the (101) peak. **b,** RAMAN spectrum of the TG composite that clearly shows the D and G peaks (graphene).

**Supplementary Fig .2**

**Supplementary Fig. 2|**UV−vis diffuse reflectance spectra of TiO_2_nanoboxes and TG composites.

**Supplementary Fig .3**

**Supplementary Fig 3. |**XRD patterns for (i) TiO_2_ hollow nanoparticles, (ii) Fe_3_O_4_@SiO_2_core-shell nanocomposites and (iii) TSFG composites.

**Supplementary Fig .4**


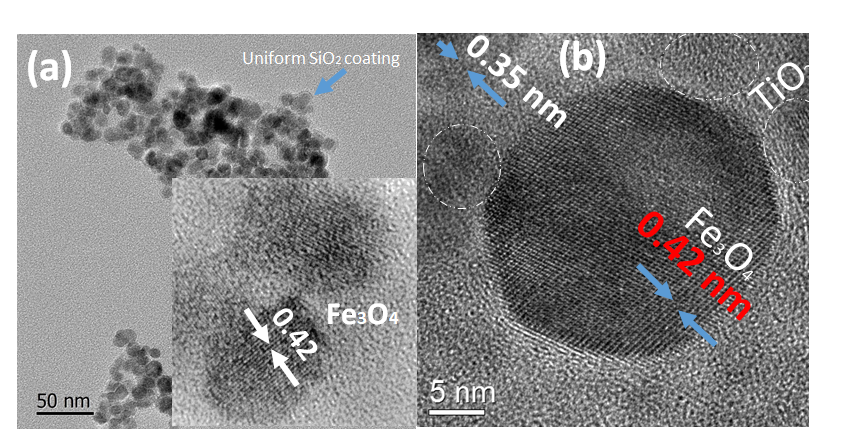


**Supplementary Fig 4. |a.** TEM of Fe_3_O_4_@SiO_2_ nanoparticles with inset showing the HRTEM image, **b.**HRTEM of Fe_3_O_4_@SiO_2_ and TiO_2_ nanoboxes.

**Supplementary Fig .5**

**Supplementary Fig. 5 |a.**Magnetization curves for the Fe_3_O_4_, **b.**Fe_3_O_4_@SiO_2_ and**c.**TFSG composites at room temperature.

**Supplementary Fig .6**

**Supplementary Fig. 6 |**The ^•^OH radical amount-related fluorescence spectra of **a.**TiO_2_nanoboxes,b. TFSG, and **c.** TG composites.

**Supplementary Fig .7**


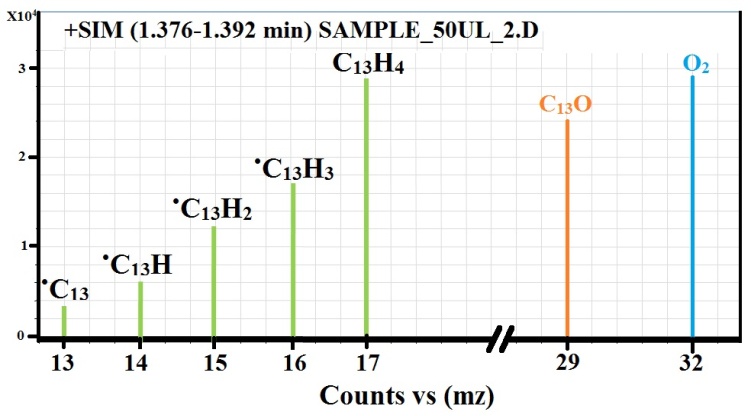


**Supplementary Fig. 7 |**m/z values for tested productions of the TG after photocatalytic reduction of isotopic ^13^CO_2_ under visible-light irradiation.

**Supplementary information 8**

**Quantum efficiency**

Here we have calculated the quantum efficiency of TiO_2_, TGF and TG for CO_2_ reduction at λ = 400 nm: The catalyst mixture was irradiated by a 300 W Xe lamp. The average incident irradiation was determined to be 2.75 mW/cm^2^ by Newport (Oriel instrument USA model 91150V ser. No 391/0118) and area was 3.5 cm^2^. The amount of CH_4_ gas produced for TiO_2_, TGF and TG were 0.3, 2.01 and 3.17 μmol, respectively. All the calculations are given below.

$$\boldsymbol{N}\boldsymbol{=}\frac{\boldsymbol{E\lambda}}{\boldsymbol{hc}}\boldsymbol{=}\frac{\boldsymbol{2.7}\boldsymbol{\times}\boldsymbol{10}^{\boldsymbol{-}\boldsymbol{3}}\boldsymbol{\times}\boldsymbol{3.5}\boldsymbol{\times}\boldsymbol{420}\boldsymbol{\times}\boldsymbol{10}^{\boldsymbol{-}\boldsymbol{9}}\boldsymbol{\times}\boldsymbol{8}\boldsymbol{\times}\boldsymbol{3600}}{\boldsymbol{6.626}\boldsymbol{\times}\boldsymbol{10}^{\boldsymbol{-}\boldsymbol{34}}\boldsymbol{\times}\boldsymbol{3}\boldsymbol{\times}\boldsymbol{10}^{\boldsymbol{-}\boldsymbol{8}}}$$

$$\boldsymbol{N}\boldsymbol{=5.85}\boldsymbol{\times}\boldsymbol{10}^{\boldsymbol{20}}$$

$$\boldsymbol{QE}\boldsymbol{=}\frac{\left( \boldsymbol{2}\boldsymbol{\times}\boldsymbol{thenumberofH} \right)\boldsymbol{+}\left( \boldsymbol{2}\boldsymbol{\times}\boldsymbol{thenumberofCO} \right)\boldsymbol{+}\left( \boldsymbol{8}\boldsymbol{\times}\boldsymbol{thenumberof}\boldsymbol{CH}_{\boldsymbol{4}} \right)\boldsymbol{moleculesproduced}}{\boldsymbol{thenumberofincidentphotons}}$$

$$\boldsymbol{QE}_{\boldsymbol{TiO}_{\boldsymbol{2}}}\boldsymbol{=}\frac{\left( \boldsymbol{2}\boldsymbol{\times}\boldsymbol{0.2} \right)\boldsymbol{+}\left( \boldsymbol{2}\boldsymbol{\times}\boldsymbol{0.05} \right)\boldsymbol{+}\left( \boldsymbol{8}\boldsymbol{\times}\boldsymbol{0.3} \right)\boldsymbol{\times}\boldsymbol{10}^{\boldsymbol{-}\boldsymbol{6}}\boldsymbol{\times}\boldsymbol{6.02}\boldsymbol{\times}\boldsymbol{10}^{\boldsymbol{23}}}{\boldsymbol{5.85}\boldsymbol{\times}\boldsymbol{10}^{\boldsymbol{20}}}\boldsymbol{\times}\boldsymbol{100 \%}$$

$$\boldsymbol{∎ QE}_{\boldsymbol{PCN}}\boldsymbol{= 0.30\%}$$

$$\boldsymbol{QE}_{\boldsymbol{TGF}}\boldsymbol{=}\frac{\left( \boldsymbol{2}\boldsymbol{\times}\boldsymbol{1} \right)\boldsymbol{+}\left( \boldsymbol{2}\boldsymbol{\times}\boldsymbol{0.7} \right)\boldsymbol{+}\left( \boldsymbol{8}\boldsymbol{\times}\boldsymbol{2.01} \right)\boldsymbol{\times}\boldsymbol{10}^{\boldsymbol{-}\boldsymbol{6}}\boldsymbol{\times}\boldsymbol{6.02}\boldsymbol{\times}\boldsymbol{10}^{\boldsymbol{23}}}{\boldsymbol{5.85}\boldsymbol{\times}\boldsymbol{10}^{\boldsymbol{20}}}\boldsymbol{\times}\boldsymbol{100 \%}$$

$$\boldsymbol{∎ QE}_{\boldsymbol{TGF}}\boldsymbol{= 2.01 \%}$$

$$\boldsymbol{QE}_{\boldsymbol{TG}}\boldsymbol{=}\frac{\left( \boldsymbol{2}\boldsymbol{\times}\boldsymbol{1.1} \right)\boldsymbol{+}\left( \boldsymbol{2}\boldsymbol{\times}\boldsymbol{0.9} \right)\boldsymbol{+}\left( \boldsymbol{8}\boldsymbol{\times}\boldsymbol{3.17} \right)\boldsymbol{\times}\boldsymbol{10}^{\boldsymbol{-}\boldsymbol{6}}\boldsymbol{\times}\boldsymbol{6.02}\boldsymbol{\times}\boldsymbol{10}^{\boldsymbol{23}}}{\boldsymbol{5.85}\boldsymbol{\times}\boldsymbol{10}^{\boldsymbol{20}}}\boldsymbol{\times}\boldsymbol{100 \%}$$

$$\boldsymbol{∎ QE}_{\boldsymbol{TG}}\boldsymbol{= 3.02 \%}$$

**Supplementary information 9**

**Experimental**

Materials

NH_4_OH, ethylene glycol, FeCl_3_.6H_2_O, FeCl_2_.4H_2_O, Titanium *n*-tetrabutoxide (TBT), ethanol, titanium tetrachloride, tert-butylalcohol, 4-tertbutylpyridine (4-TBP), 1-methyl-3-propyl imidazolium iodide (PMII), Acetic acid (AcOH), hydrofluoric acid (HF), ethyl cellulose and tetraethyl orthosilicate were all obtained from Rich Joint Chemical Reagent Co., Ltd. (Shanghai, China) and graphene oxide from XFNANO Company Nanjing. All the chemical reagents were used without further purification.

Preparation of hollow TiO_2_nanoboxes

Anatase hollow nanoboxes were prepared by hydrothermal method. 1 mL of TBT was slowly introduced into 30 mL of AcOH to produce a white solution via magnetic stirring. After keeping under vigorous stirring for 30 minutes 100 µL of HF was added in the solution. This white mixture was then transferred into a 50 mL Teflon-lined stainless steel autoclave, sealed and kept in an oven at 160 °C for 6 h. The final product was centrifuged at 3500 rpm for 5 min. The white precipitate was washed with water then centrifuged again and finally dried at 80 °C for 5 hours. These nanoboxes were calcined for 3 h at 500 °C to obtain pure anatase TiO_2_ phase.

Preparation of Fe_3_O_4_@SiO_2_ magnetic nanoparticles:

For the preparation of Fe_3_O_4_ nanoparticles FeCl_3_.6H_2_O and FeCl_2_.4H_2_O salts were mixed with mass ratio of 2:1 in 30 mL deionized water under N_2_ gas bubbling and magnetic stirring. 3 mL of aqueous ammonia solution (28 wt. %) was added drop-wise to get pH = 10. The Fe_3_O_4_ nanoparticle precipitates were separated by magnet from the solution and then washed with deionized water, acetone and ethanol. Finally, the particles were dried at 50 ^o^C for 5 hours under vacuum.

The modified Stober method was used for preparing core-shell structure of Fe_3_O_4_@SiO_2_ nanoparticles. Firstly, 0.1 gram of magnetite nanoparticles were dispersed in the solution of 20 mL deionized water and 80 mL ethanol. Then, 0.2 mL of (TEOS) in 50 mL ethanol and 6 mL NH_4_OH (28 wt.%) was added to the solution. The reaction was then allowed to proceed at room temperature under magnetic stirring for 4 h. The product was separated from the solution by external magnet and then washed several times with water. After washing, the final product was dried at 40^o^C under vacuum.

Preparation of TFSG composites:

TiO_2_ nanoboxes, Fe_3_O_4_@SiO_2_ and graphene oxide with weight ratios of 85%, 5% and 10%, respectively, were mixed and sonicated for 60 minutes. 15 mL of the mixed solution was poured into glass-tube, sealed and heated in microwave reactor (NOVA-2S) at 190 ^o^C for 30 minutes. After that the microwave reactor was cooled down to 70 ^o^C. The precipitate was collected and washed with deionized water and ethanol, then dried at 50 ^o^C for 6 hours. The final product of TiO_2_-(Fe_3_O_4_@SiO_2_)@rGO hybrid was defined as TFSG composites in this work. Similarly, we prepared TG composites (TiO_2_@rGO).

Structure and defects characterization

Positron annihilation lifetime spectra (PALS) were measured using a conventional ORTEC-583 fast–fast coincident system at room temperature. The coincidence spectrometer used had a prompt time resolution of 270 ps (FWHM) for the γ-rays from a ^60^Co source selected under the experimental conditions. The sample powder was pressed into a disk (diameter: 10.0 mm, thickness: 1.0mm). A 30µci ^[^[^20^](#_ENREF_20)^]^Na positron source was sandwiched between two identical sample disks. Each spectrum contained 1.0×106 for PATFIT. The positron lifetime spectrum containing 106 counts was analyzed by the PATFIT program to get several lifetime components. The summary of data is given below.

Powder X-ray diffraction was obtained (XRD, D-MAX2500) to identify the crystal phase. Cu target (CuKα=1.5418 Å) with 2*θ* range from 20° to 80° was used. The particle size and morphology of the powder were observed by transmission electron microscopy with JEM-2100F operating at 200 kV. The UV-vis spectra of the samples were recorded in the range from 200 to 800 nm by Perkin Elmer UV Lambda 900 spectrophotometer. The photocurrent response and electrochemical impedance spectra measurements were taken with an electrochemical system (CHI-660C) and three quartz cells with 0.1 M Na_2_SO_4_ electrolyte solution. The TiO_2_nanoboxes, TG composites and TGF composites electrodes on FTO were used as the working electrode (WE). The spin coating method was employed to make film on the WE, and sintered in Argon atmosphere for two hours at 200 ^o^C. The defect densities of the samples were measured by the X-ray photoelectron spectrum (XPS) (PHI-5000, US). The binding energies were calibrated by the C1s peak at 284.6 eV. The magnetic properties were tested by a physical property measurement system (PPMS-9, Quantum Design Co.).

Electrochemical characterization

The electrochemical impedance spectroscopy (EIS) measurements were conducted at open circuit potential using CS350 Electrochemical Workstation in the frequency range from 0.01 Hz to 100 kHz by superimposing an AC voltage of 10 mV amplitude. The electron paramagnetic resonance (EPR) spectrometer settings in a standard photochemical experiment were microwave frequency, ~9.424 GHz; microwave power, 10.53 mW; center field, 335.6 mT; sweep width, 8–16 mT; gain, 1 × 105 to 1 × 106 ; modulation amplitude, 0.05–0.1 mT; scan, 20 s; time constant, 10.24 ms. The g-values (±0.0001) were determined using a built-in magnetometer.

Evaluation of CO_2_ conversion to CH_4_

Photocatalytic CO_2_ reduction was carried out in a closed gas circulation-evacuation reactor. Before the photocatalytic reduction, the following three steps were performed. Firstly, catalyst powders (50 mg) were dispersed in a water solution (20 mL) with 10 vol% triethanol amine (TEOA, pH=9), which serves as an electron donor, in the reactor. Then, the reactor was evacuated and refilled with pure CO_2_ gas for three times to remove the air inside. Finally, the reactor was filled with CO_2_ at a pressure of 1.01 bar. The used photo-catalysts were equilibrated in the CO_2_/H_2_O system for one hour. During the photo-catalytic reduction, the reactor was irradiated with Xe-lamp (300 W) under stirring. The produced gas was detected by a gas chromatography (GC 7900) with both TCD and FID detectors.

Evaluation of photocatalytic H_2_ production

The photo-catalytic H_2_ generation was performed in a pyrex top-irradiation reaction vessel with a closed glass gas circulation system (Lab-solar III AG, Beijing Perfect light Technology Co. Ltd, China). The visible light source came from a 300 W, Xe-lamp (PLSSXE 300/300UV, Beijing Perfect light Technology Co. Ltd, China) with both UV(380 nm filter) and visible light (a 420 nm cut-off filter). The incident light intensity was 100 mW/cm^2^ measured by a PL-MW2000 Photo radiometer (Beijing Perfect light Technology Co. Ltd, China). A 20 mg catalyst was dispersed into an aqueous solution (100 mL) containing triethanolamine (10 vol.%), and 3 wt.% Pt was deposited on the surface of the sample by *in-situ* photo-reduction using H_2_PtCl_6_ as a precursor. The closed gas circulation system connecting to the solution was vacuumed before the photo-catalytic reaction. The released gases were analyzed by a gas chromatography (GC7806, Beijing Shiweipuxin Analytical Instruments Co. Ltd, China) equipped with a thermal conductive detector and a 5Å molecular sieve column. N_2_ was used as the carrier gas.

**Supplementary information D1**

The C1s XPS spectra indicate the existence of chemical binding between TiO_2_ and rGO (Fig. 1a and b). Compared with the blank GO, the two new peak at 285.5 eV are ascribed to Ti-O-C^1-4^. Therefore there is covalent bonding between the TiO_2_ and rGO.


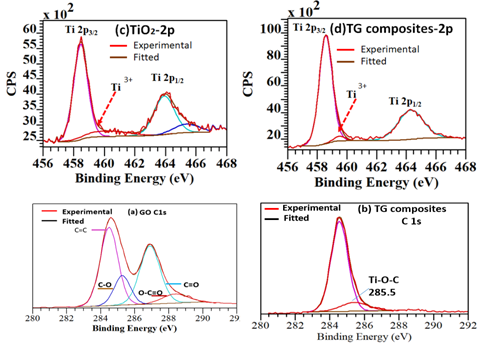


**Fig. 1 |**XPS spectra of C 1s spectrum GO (a), TG composites (b), Deconvoluted XPS spectra of Ti2p peak showing Ti^3+^ and Ti^4+^ states of TiO_2_nanoboxes and (c) TG composites(d).

For further investigation, the XPS was studied to understand the chemical composition and the Ti oxidation-state of TiO_2_ nano-boxes after microwave treatment with GO. The variation in the chemical state of elements ‘O’ and ‘Ti’ was analyzed in detail. The high-resolution XPS spectrum (Fig. 1c) of TiO_2_ shows, the doublet Ti2p_3/2_ (binding energy 458.5 eV) and Ti2p_1/2_ (464.3 eV)’ arising from the spin orbit-splitting. These peaks are consistent with Ti^4+^ in TiO_2_ lattice^5^. These peaks also reveal 5.46 eV binding energy difference (or splitting) between Ti2p_3/2_ and Ti2p_1/2_, which indicates the presence of Ti^4+^ state. This splitting was increased to 5.75 eV in case of TFSG composites. In addition, the shoulder Ti2p_1/2_ at binding energy 460.2 eV corresponds to Ti^3+^ in Ti_2_O_3_.^6^ After microwave treatment of TiO_2_ with GO, the high-resolution XPS spectrum (Fig. 4d) shows a slight position shift along with a variation in the peaks area. The peaks in the TG composites are now located at binding energies 458.6 (T_i_2p_3/2_) and 464.3 eV (T_i_2p_1/2_). The shift of these peaks indicates an influence of GO hybridization on the electronic states. The decrease (three times) in the area of Ti^3+^ peak indicates that Ti_2_O_3_ amount was decreased. The increased area of Ti^4+^ indicates Ti-O-C formation in the TiO_2_ lattice. Whereas the wide and broad peaks 500-900 cm^−1^ are attributed to the stretching vibration of Ti-O-Ti and Ti-O-C bonds.^8^

**Fig. 2 |**FTIR spectra of TiO_2_ and TG composites

Furthermore, peak strength of TiO_2_/rGO nanocomposites is lower than the TiO_2_/GO nanocomposites and some of the peaks even disappear. From the FTIR spectrum of TiO_2_/rGO nanocomposites, few peaks indicating the oxygen containing functional groups can be seen after reduction. These oxygen-containing functional groups demonstrate the successful preparation of GO and provide anchoring sites for the adsorption of heavy metal ions on the TG nanocomposites. It had been confirmed that the oxygen-containing functional groups played an important role in the adsorption of heavy metal ions.^8-10^

[1] Xing, M., Shen, F., Qiu, B. & Zhang, J. Highly-dispersed Boron-doped Graphene Nano sheets Loaded with TiO_2_ Nanoparticles for Enhancing CO_2_ Photoreduction. Sci. Rep. 4, 6341–6347 (2014).

[2]Zhang, J., Pan, C., Fang, P., Wei, J. &Xiong, R. Mo 1 C codoped TiO2 using thermal oxidation for enhancing photocatalytic activity. ACS Appl. Mater. Interfaces 2, 1173–1176 (2010).

[3]Zhang, Y. & Pan, C. TiO_2_/graphene composite from thermal reaction of graphene oxide and its photocatalytic activity in visible light. J. Mater. Sci. 46, 2622–2626 (2011).

[4]Yang, J., Bai, H., Jiang, Q. & Lian, J. Visible-light photocatalysis in nitrogen– carbon-doped TiO_2_ films obtained by heating TiO_2_ gel–film in an ionized N_2_ gas. Thin Solid Films 516, 1736–1742 (2008).

[5] Sanjinés, R. et al. Electronic structure of anatase TiO_2_ oxide. J. Appl. Phys. 75, 2945–2951 (1994).

[6]Bert, I., Mohai, M., Sullivan, J. L. & Saied, S. O. Surface characterization of plasma-nitrided an XPS study titanium: Appl. Surf. Sci. 84, 357–371 (1995).

[7]Kato, S. et al. Quantitative depth profiling of Ce^3+^ in Pt/CeO_2_ by in situ high-energy XPS in a hydrogen atmosphere. Phys. Chem. Chem. Phys. 17, 5078–5083 (2015).

[8]Reduced graphene oxide and Ag wrapped TiO_2_photocatalyst for enhanced visible light photocatalysis, APL MATERIALS 3, 104503 (2015).

[9] S. Umrao, S. Abraham, F. Theil, S. Pandey, V. Ciobota, P. K. Shukla, C. J. Rupp, S. Chakraborty, R. Ahuja, and J. Popp,RSC Adv. 4(104), 59890–59901 (2014).

[10] Y. Liu, K. Chen, M. Xiong, P. Zhou, Z. Peng, G. Yang, Y. Cheng, R. Wang, and W. Chen, RSC Adv. 4(82), 43760–43765(2014).

**Supplementary information D2**

These defects were tested by the HF addition experiments. The HF is capable to remove oxygen and cause oxygen vacancies. Defects were introduced into TiO_2_ by changing the amount of hydrofluoric acid (HF, Fig. 1, 2). The comparison showed defect introduction by the HF (0, 50 µL) changed the shape of EPR, introduced extra peaks (less smooth EPR spectra), lowered the signal and modified the g value of TiO_2_. ^1,2,3,4^

**Fig. 1 |** EPR of pristine TiO_2_ (HF = 0 µL).

**Fig. 2 |** EPR of HF treated TiO_2_ (HF = 50 µL).

[1] X. Chen, L. Liu, P. Y. Yu and S. S. Mao, Science, 2011, 331, 746.

[2] A. Naldoni, M. Allieta, S. Santangelo, M. Marelli, F. Fabbri, S. Cappelli, C. L. Bianchi, R. Psaro and V. Dal Santo, J. Am. Chem. Soc., 2012, 134, 7600.

[3] H. Liu, H. T. Ma, X. Z. Li, W. Z. Li, M. Wu and X. H. Bao, Chemosphere, 2003, 50, 39. [4] Reduced TiO_2_-Graphene Oxide Heterostructure As Broad Spectrum Driven Efficient Water-Splitting Photocatalysts, ACS Appl. Mater. Interfaces 2016, 8, 8536-8545.

**Supplementary information D3**


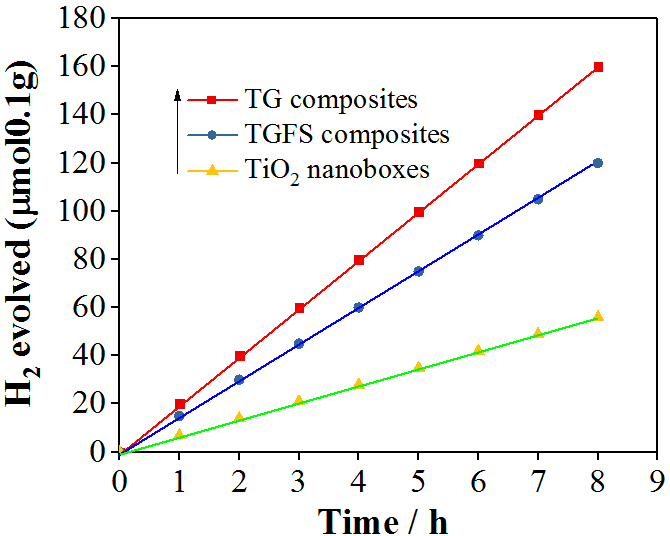
Stability of samples was tested by the H_2_ evolution for 8 h light irradiation. The Fig. 1 shows comparison of time dependent H_2_ evolution for the TiO_2_, TG and TFSG composites. The standard solar light illumination for 8 hours, the hydrogen evolution kept increasing, demonstrating the robust photo-catalytic performance. This observation indicates stable and active photo-catalyst for long term water splitting. The rate of H_2_ evolution followed the following order: TG composites > TFSG composites > TiO_2_ nano-boxes respectively.

**Fig. 1 |** Time courses of H_2_ evolution resulting from the reaction of water by TiO_2_ nanoboxes, TG composites and TFSG composites irradiated with UV-visible light.


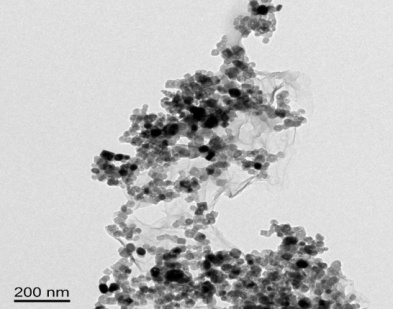


Fig. 2 | TEM of TG composites after 5 repeated cycles (5 h light irradiation).

After conversion of CO_2_ to CH_4_ for 5h, samples were collected and washed several times. Fig 2 shows TEM result after 5 h light irradiation. These results show that TG composites are stable and not reduced under light irradiation.
